# Dendrobium Officinale Polysaccharides Protect against MNNG-Induced PLGC in Rats via Activating the NRF2 and Antioxidant Enzymes HO-1 and NQO-1

**DOI:** 10.1155/2019/9310245

**Published:** 2019-06-04

**Authors:** Yi Zhao, Youzhi Sun, Gaoyu Wang, Shucao Ge, Hongning Liu

**Affiliations:** ^1^Research Center for Differentiation and Development of Basic Theory of Traditional Chinese Medicine, Jiangxi University of Traditional Chinese Medicine, Nanchang 330004, China; ^2^Jiangxi Province Key Laboratory of TCM Etiopathogenisis, Nanchang 330004, China; ^3^School of Basic Medical Sciences, Jiangxi University of Traditional Chinese Medicine, Nanchang 330004, China

## Abstract

Dendrobium officinale polysaccharides (DOP) are the main effective ingredient in Dendrobium officinale. Nuclear factor erythroid 2-related factor 2 (NRF2) signaling is regarded as an important way to mitigate the effects of reactive oxygen species (ROS) damage and inhibit gastric cancer progress. This study introduces a previously unknown effect of DOP on precancerous lesions of gastric cancer (PLGC). The mechanism discussed herein is based on the NRF2 signal pathway as well as its downstream antioxidant enzymes heme oxygenase-1 (HO-1) and NADPH quinone oxidoreductase-1 (NQO-1). DOP was prepared by the alcohol deposition method, and its molecular weight was determined using High-Performance Gel-Permeation Chromatography (HPGPC). Sixty male rats were randomly divided into five groups: normal control group (NC), PLGC model group (PLGC), model treated with low dose (2.4 g/kg) of DOP (L-DOP), model treated with middle dose (4.8 g/kg) of DOP (M-DOP), and model treated with high dose (9.6 g/kg) of DOP (H-DOP). DOP was orally administered to rats for 15 consecutive days prior to the start of a seven-month course of 1-methyl-3-nitro-1-nitrosoguanidine (MNNG) exposure. Histological evaluation was observed by hematoxylin and eosin (HE) and alcian blue/periodic acid-Schiff (AB-PAS) staining. Alanine aminotransferase (ALT), aspartate transaminase (AST), serum creatinine (Scr), serum uric acid (UA), blood urea nitrogen (BUN), and HE staining were detected for liver and kidney function. The level of 8-hydroxy-deoxyguanosine (8-OHdG) in serum was detected by kits. The NRF2 protein expression was detected by immunohistochemistry, and western blotting was utilized to compare differential protein expression levels among cytoplasmic and nuclear cell fractions. Expression levels of antioxidant enzymes heme oxygenase 1 (HO-1), Glutamate-Cysteine Ligase Catalytic Subunit (GCLC), Glutamate-Cysteine Ligase Modifier Subunit (GCLM), and NAD(P)H: quinone oxidoreductase-1 (NQO-1) were analyzed by reverse transcriptase polymerase chain reaction (RT-PCR); furthermore, the protein expression of NRF2, HO-1, and NQO-1 was detected by western blotting. The results showed that the average content of DOP is 83%, and its molecular weight is mainly contained within 3500 and 1000000. The H-DOP experimental group exhibited noticeable weight gain after seven months, reduced intestinal metaplasia, and made the atypical hyperplasia to be kept in moderate or mild degree. Data also showed DOP to be capable of decreasing levels of ALT, UA, and BUN, all of which had been elevated following the appearance of MNNG-induced PLGCs. DOP was also seen to reduce the expression of 8-OHdG and promote the expression of NRF2 in the gastric mucosa. Furthermore, RT-PCR and western blotting results showed that DOP upregulated the gene and protein expression of HO-1 and NQO-1. These findings show that DOP prevents MNNG-induced PLGC along with subsequent liver and kidney damage. The protective effects of DOP are associated with the reduction of 8-OHdG levels as well as the activation of the NRF2 pathway and its related antioxidant enzymes, HO-1 and NQO-1.

## 1. Introduction

Gastric cancer is the fifth most common cancer and is the third leading cause of cancer deaths in the world [1]. Development of gastric cancer is a multistep process, including normal epithelia to nonatrophic gastritis (NAG), multifocal atrophic gastritis (MAG) without intestinal metaplasia, intestinal metaplasia (IM), dysplasia, and cancer. Precancerous lesions of gastric cancer (PLGC), including intestinal metaplasia and atypical hyperplasia, is regarded as an ideal stage for early detection and prevention [2–4].

The main causes of gastric cancer are diet, alcohol, tobacco, and Helicobacter pylori infection, all of which may cause ROS production and DNA damage [5]. Increased reactive oxygen species and oxidative stress could damage gastric mucosa and lead to cancer [6].The genes encoding NRF2 and KEAP1 play an important role in the induction of antioxidant enzymes against oxidative stress [7, 8]. NRF2 signaling is considered an important molecular target for cancer prevention, and some traditional Chinese herbal medicines and dietary phytochemicals are thought to target NRF2-mediated pathways of oxidative stress and anti-inflammatory responses to prevent carcinogenesis [9–11].

D. officinale Kimura et Migo (D. officinale) (*Tiepi Shihu* in Chinese) belongs to genus Dendrobium [12] and displays anticancer effects on MCF cells, HCT-116 cancer cells, and HepG2 liver cancer cells in vitro [13–15]. Researchers also discovered that D. officinale exhibited preventive effects on the formation of lung metastases and colon carcinogenesis in mice [16, 17]. Our research group has found that D. officinale extraction (DOE) can prevent gastric carcinogenesis in rats through upregulating Bax and downregulating such factors as antiapoptotic B cell lymphoma 2 (Bcl-2), epidermal growth factor (EGF), epidermal growth factor receptor (EGFR), and sphingosine-1-phosphate (S1P) [18, 19]. Further analysis revealed that Dendrobium officinale extracts could regulate the levels of 8-OHdG, superoxide dismutase (SOD), malondialdehyde (MDA), and glutathione peroxidase (GSH-PX) in plasma and cytokines related to carcinogenesis [20]. DOP, the main effective part in Dendrobium officinale, had been reported to harbor anticancer effects on gastric cancer cells and protective effects on experimental gastric ulcers in mice [21, 22]. This study explores, for the first time, the ability of DOP to prevent PLGCs via a mechanism based on NRF2 and important downstream antioxidant enzymes such as HO-1 and NQO-1.

## 2. Materials and Methods

### 2.1. Animals

Male Wistar rats (70 to 90 g) were purchased from Beijing Vital River Laboratory Animal Technology Co. Ltd. (certificate Number SCXK 2015-0002, Beijing, China). The rats were fed in the standard experimental conditions (room temperature 23 ± 1°C, relative humidity 55 ± 5%) with a 12 h light/dark cycle and received food and water. Before the experiment, the rats were adapted to the experimental environment for 1 week. The protocol for these experiments was approved by the international ethical guidelines and the Institutional Animal Care and the Animal Ethics Committee of Jiangxi University of Traditional Chinese Medicine.

### 2.2. Drugs and Reagents

Dendrobium officinale extraction (DOE) was purchased from Zhejiang Shou Xian Valley Medical Limited by Share Ltd. (Jinhua, Zhejiang, China), with the voucher specimen number of SXG150709. The fresh D. officinale added a 20-fold volume of water and extracted three times by stirring for 2 hours one time, then evaporated and formed the water extraction (DOE). The quality control of DOE had been studied before [20]. Glucose was purchased from Nanchang Beta Biotech Co. Ltd. (Nanchang, Jiangxi, China). Dextrans (T3, T10, T50, T70, T110, T200, and T2000) were purchased from Shanghai Jinsui Biotechnology Co. Ltd. (Shanghai, China). The gel chromatography column used (TSK-gel, G4000PWXL, 7.8 mm × 300 mm) was obtained from TOSOH (TOSOH, Tokyo, Japan). Rat 8-OHdG enzyme-linked immunosorbent assay (ELISA) kits were obtained from Wuhan USCN Business Co. Ltd. (Wuhan, Hubei, China). Total RNA Extraction Kit and Reverse Transcription PCR Kit were the products of Promega Corporation (Promega, WI, USA). FastStart Essential DNA Green Master was the product of Roche (Roche, Basel, Switzerland). NE-PER Nuclear and Cytoplasmic Extraction Reagents and Goat anti-Rabbit IgG (H + C) were purchased from Thermo Fisher Scientific (Thermo, MA, USA). Anti-NRF2 antibody (LOT number: C0271), anti-HO-1 antibody (LOT number: GR284419-8), and anti-actin (LOT number: GR105851-10) were purchased from Abcam Company (Abcam, Cambridge, Britain). Anti-Histone H3 (LOT number: 0009) was purchased from Cell Signaling Technology Inc. (Danvers, MA, USA). Anti-NQO-1 antibody was purchased from Santa Cruz Biotechnology Inc. (Santa Cruz, CA, USA). The Polyvinylidene Fluoride (PVDF) membrane was purchased from Millipore (Millipore, MA, USA).

### 2.3. Preparation and Content of DOP

The 50 g water extraction was dissolved in 2.5 L water and then mixed with 95% alcohol at a ratio of 1:  5. After 24 hours, the mixture was centrifuged at 4000 rpm for 15 min and the supernatant was removed. This process was repeated twice. The precipitate was then washed sequentially with ethanol and acetone then fully dissolved with 80% ethanol. In the end, the precipitate was dried to dry powder and formed crude DOP. Crude DOP was dissolved in water, and 1/2 sample volume of savage reagent was added. The mixture was shook vigorously for 10 min and then refrigerated at 4°C overnight. This process was repeated two times. The polysaccharide content was determined by the phenol-sulfuric acid method. The protein content of DOP was determined by BCA assay using bovine serum albumin (BSA) as standard.

### 2.4. Determination of DOP Molecular Weight by High-Performance Gel-Permeation Chromatography (HPGPC)

The analysis was performed at 26°C with the mobile phase 0.3% Na_2_SO_4_ solution, a high-performance gel column, and a refractive index detector (LDC) (Agilent RID-G1362A) was used in a series. Molecular weight standards of dextran (T3, T10, T50, T70, T110, T200, and T2000) as well as the sample itself were all used at a concentration of 5 mg/mL. The standards and the sample were all filter-sterilized through a 0.22 *μ*m membrane filter using a predetermined flow rate of 0.5 mL/min. Injection volume was 20 *μ*L. The calculation of average molecular weight was based on the retention time of the standards [23].

### 2.5. Inhibition Effects of DOP on PLGC in Rats

Sixty male rats were randomly divided into five groups: normal control group (NC), PLGC model group (PLGC), low dose (2.4 g/kg) of DOP (L-DOP), middle dose (4.8 g/kg) of DOP (M-DOP), and high dose (9.6 g/kg) of DOP (H-DOP). The PLGC model group was given 150 *μ*g/mL of MNNG in drinking water for 7 months and given 0.1 mL of 10% NaCl once weekly during the initial 20 weeks [24–26]. During the pretrial period, rats were kept and fed in groups in the laboratory at room temperature (22-24°C). DOP were administered to rats 15 days before inducing the PLGC model and lasted 7 months until the end of experiment. The body weight of each rat was measured once a week. After seven months, all animals were sacrificed by intraperitoneal injection of thiopental. The serum from the inferior vena cava was collected in a tube and centrifuged at 3000 rpm at 4°C for 10 min. This was subsequently used to assess liver and kidney function as well as 8-OHdG levels. The stomach was cut open on ice along the greater curvature and divided into three parts. One was fixed in 4% paraformaldehyde (PFA) solution for histological study and AB-PAS staining. Another was stored at −80°C for western blot analysis, and the final part was kept in a RNA preservation solution for RT-PCR analysis.

### 2.6. Histological Evaluation

Morphological changes were analyzed with HE staining. Stomach tissue was fixed in 10% neutral-buffered formalin overnight and then subject to alcohol and xylene dehydration, respectively. Dehydrated samples were embedded in paraffin, cut into sections (4 *μ*m), and then stained with hematoxylin and eosin. Additionally, AB-PAS staining was performed according to the manufacturer's instructions in order to detect different types of intestinal metaplasia.

### 2.7. Pathological Observation and Assay of Serum ALT, AST, Scr, UA, and BUN Levels from Liver and Kidney Tissues

The activity of ALT, AST, Scr, UA, and BUN in serum was determined by the standard methods with the Automatic Biochemistry Analyzer (ACTA, Italia). HE staining was used to conduct the pathological analysis of liver and kidney tissues.

### 2.8. Analysis of 8-OHdG in Serum

The concentrations of 8-OHdG in serum were measured using rat 8-OHdG ELISA kits according to the manufacturer's instructions. Briefly, biotinylated antibody reagent was added to 96-well plates followed by the supernatants of homogenized serum. Plates were then incubated at 37°C in CO_2_ for 2 h. After washing with phosphate-buffered saline (PBS), streptavidin-horseradish peroxidase (HRP) solution was added, and the plate was incubated for 30 min at room temperature. The absorbance was measured at 450 *μ*m using a microplate reader.

### 2.9. Immunohistochemical Analysis of NRF2

Four *μ*m thick sections were dewaxed with xylene and hydrated using sequential ethanol washes (100, 95, 85, and 75%), ending with a distilled water wash. Antigen retrieval was performed by heating sections in 0.01 M sodium citrate buffer (pH 6.0). Tissue slides were incubated overnight with NRF2 antibody (dilution 1: 400) at 4°C prior to introduction of secondary antibodies. Sections were incubated with 3,3′diaminobenzidine (DAB) to produce a brown product and counterstained with hematoxylin. The positive staining was evaluated. Five fields of view were randomly selected for each slice under a 400-fold microscope. Image-Pro Plus 6.0 software was used to conduct mean density analysis.

### 2.10. Analysis of NRF2 Protein Expression in Cytoplasmic and Nuclear Cell Fractions

Western blot techniques were employed in analyzing protein extracts collected from stomach tissues for NFR2 expression. This was done primarily to compare the differential expression between cytoplasmic and nuclear protein extracts. Histone H3 and *β*-actin antibodies served as loading controls for nuclear and cytoplasmic extracts, respectively. Extraction process was according to the outlined protocols of NE-PER® Nuclear and Cytoplasmic Extraction Reagents.

### 2.11. Total RNA Extraction and Quantitative Real-Time RT-PCR

Total RNA was obtained from stomach tissues using the Eastep^®^ Super Total RNA Extraction Kit (Promega) protocol. RNA purity was measured with a Nanodrop 2000 (Thermo Scientific, USA). RNA samples were then reverse-transcribed into cDNA using the GoScript™ Reverse Transcription System (Promega) following the manufacturer's instructions. Primer sequences for the RT reaction (Sangon Biotech (Shanghai) Co. China) are shown in Table S1. *β*-Actin was used as a reference standard. The resulting cDNA was used as a template for RT-PCR analysis with FastStart Essential DNA Green Master (Roche, Switzerland). The conditions for RT-PCR were 95°C (10 min; preheating), 40 cycles of thermal cycle (95°C, 10 s; 55°C -60°C, 30 s; 72°C,10 s), and melt curve (95°C, 10 s; 65°C, 60 s). The relative expression for a particular gene was calculated using the Ct method, a comparative 2^ddCT method.

### 2.12. Western Blot Analysis of NRF2, HO-1, and NQO-1 Expression in Rat Stomach Tissues

The total protein samples from stomach tissues were extracted according to the manufacturer's protocols (Beyotime Biotechnology, China), and the protein content was determined using the BCA protein assay kit (Cwbiotech, Beijing, China). Protein lysates were run on a SDS-PAGE gel (10%-15%) and then transferred onto PVDF membranes (Millipore, USA). After blocking nonspecific binding sites with 5% dried skim milk, the membranes were incubated overnight at 4°C with primary antibodies. The blots were then incubated with horseradish peroxidase-conjugated secondary antibodies for 2 h at room temperature. Detection and imaging were performed using an enhanced chemiluminescence system and a ChemiDoc™ XRS Imaging System (Bio-Rad Laboratories, USA). Intensity values expressed as the relative protein expression were normalized to *β*-actin.

### 2.13. Statistical Analysis

Data were presented as mean ± standard error of mean (SEM) deviation. The statistical differences among groups were evaluated using SPSS 20.0 software (SPSS Inc., Chicago, USA) by one-way analysis of variance (ANOVA). Intergroup comparisons were followed by the least significant difference (LSD) test. Statistical significance was accepted at a value of *P* < 0.05. GraphPad Prism 5.0 software was used to make the corresponding figures.

## 3. Results

### 3.1. Preparation and Content of DOP

After UV full-wavelength scanning, the samples and standards were shown to have strong absorption at 488 nm. A standard curve was established (*Y* = 0.0825X−0.069, *R* ^2^ = 0.9889) (Figure S1). The average protein content of the three polysaccharide solutions was determined using the phenol-sulfuric acid method. Resulting sample contents were 76.59%, 86.28%, and 86.41%, and the average value was 83% (Figure S1).

### 3.2. Determination of Polysaccharide Molecular Weight by High-Performance Gel-Permeation Chromatography

**Figure 1:**
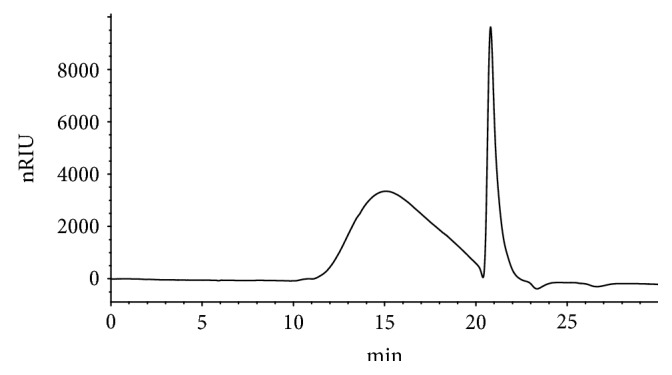
The gel chromatogram of DOP.

High-Performance Gel-Permeation Chromatography was used both to test the molecular weights of the eight different dextrans (Figure S2) and to establish a standard calibration curve based on the retention time and molecular weight of gel chromatography. The resulting regression equation is *Y* = −0.055*X*_2_ + 1.3627*X*‐2 (*R* ^2^ = 0.8736), which indicates good linearity and can be used for subsequent determination of molecular weight of samples (Figure S2). Tests for precision, repeatability, and stability were also conducted, and the RSD was 0.1862%, 0.8595%, and 0.6491%, respectively. Finally, the composition of DOP was determined to mainly consist of two kinds of polysaccharides with molecular weights of 3500 and 1000000 (Figure 1).

### 3.3. The Body Weight Change of Rats

**Figure 2:**
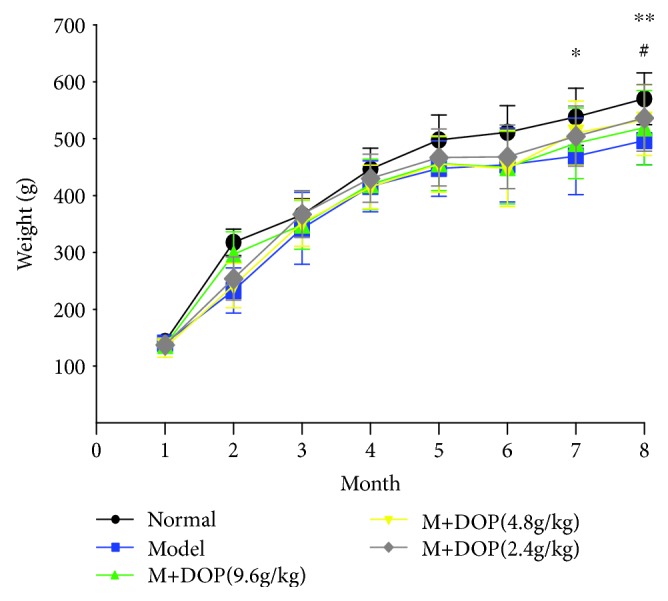
The effect of DOP on the body weight change in rats with gastric precancerous lesions (*n* = 12).

The body weight of the normal group regularly increased with each passing month. The body weight of the model group, however, increased at a noticeably slower pace, and after the sixth month, the body weight of the model group was significantly lighter than that of the normal group (*P* < 0.05, *P* < 0.01). Those which were given high levels of DOP saw elevated weight gain, with a significantly higher mean weight by the seventh month (*P* < 0.05) (Figure 2).

### 3.4. Histological Evaluation in Stomach Tissues of Rats

**Figure 3:**
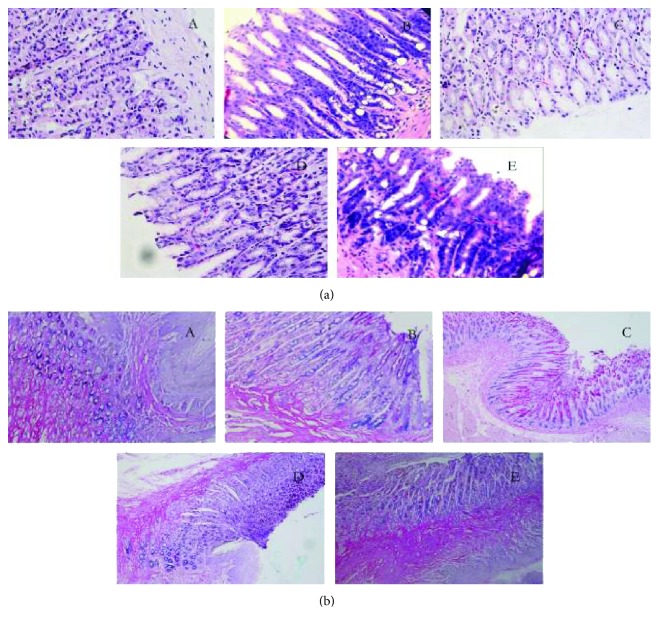
The effect of DOP on the pathological improvement of gastric carcinogenesis in rats (×400; (a) analysis by HE staining; (b) analysis by AB-PAS staining; (A) normal; (B) PLGC; (C) H-DOP; (D) M-DOP; (E) L-DOP).

The degree of gastric mucosal lesion was judged by HE histopathological examination. Compared with the normal group, the gastric mucosa was incomplete, and the dysplastic glands were significantly increased, irregularly arranged, and weakly stained. Some rats showed severe atypical hyperplasia, intestinal metaplasia, and some nuclear fission, which indicated the PLGC model was built successfully. Following administration of DOP, especially high DOP, the mucosa was basically intact, the atypical hyperplasia was moderate or mild, and the degree of intestinal metaplasia was reduced (Figure 3(a)).

AB-PAS staining, a commonly recognized method for judging the PLGC model [27], was adopted to further evaluate the establishment of the PLGC model (Figure 3(b)). In the normal group, the upper gastric mucosa was positive and dyed red by PAS staining, while the middle and bottom sections were dyed blue by AB staining. In the PLGC group, after AB staining, the middle and bottom of the gastric mucosa were positive, and the blue layer increased and thickened, indicating an acidic mucus; the upper was weakly PAS positive with only a small amount of red staining. After treatment with DOP, the positive staining of AB at the bottom was reduced, and the blue layer was also reduced and thinned. The upper showed PAS staining was positive, and the red layer increased and thickened.

### 3.5. Assay of the Serum ALT, AST, Scr, UA, and BUN Levels and the Pathological Observation of Liver and Kidney Tissues

**Figure 4:**
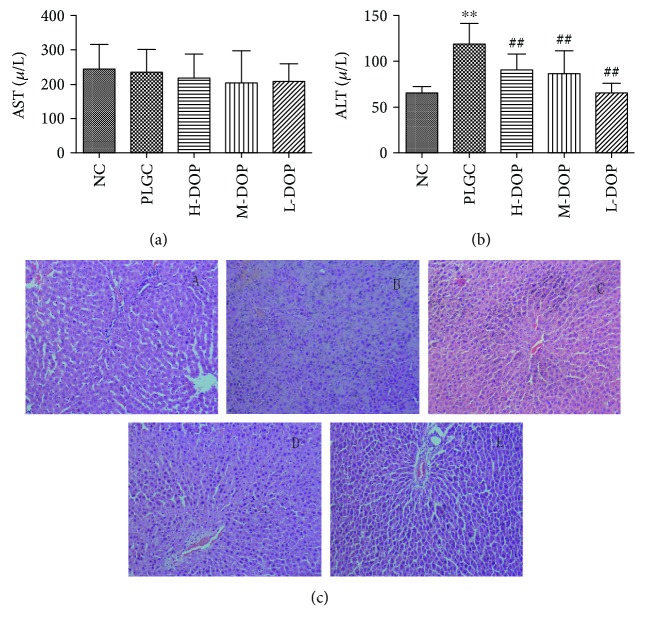
The protective effect of DOP in liver function in rats with gastric precancerous lesions ((a) AST; (b) ALT; (c) HE staining on liver tissue in rats, ×200, (A) normal; (B) PLGC; (C) H-DOP; (D) M-DOP; (E) L-DOP. ^∗∗∗^ *P* < 0.01 vs. normal group; ^##^ *P* < 0.05 vs. PLGC group).

**Figure 5:**
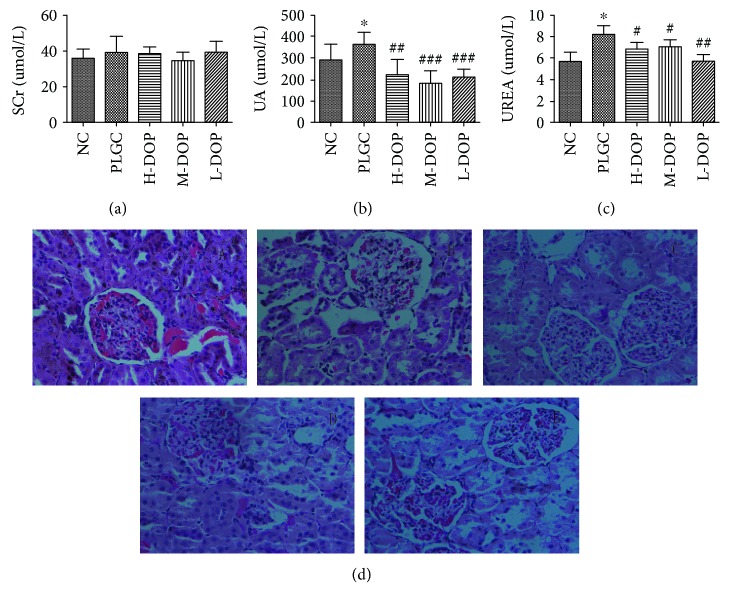
The protective effect of DOP in kidney function in rats with gastric precancerous lesions ((a) Scr; (b) UA; (c) UREA; (d) HE staining on kidney tissue in rats, ×400, (A) normal; (B) PLGC; (C) H-DOP; (D) M-DOP; (E) L-DOP. ^∗∗∗^ *P* < 0.01 vs. normal group; ^##^ *P* < 0.05 vs. PLGC group).

The data showed that AST and Scr levels were not significantly different between the groups. Under the effect of chemical carcinogen MNNG, the model group exhibited elevated ALT, UA, and BUN levels as a result of affected liver and kidney function (*P* < 0.001, *P* < 0.05, and *P* < 0.05). After treatment with high, medium, and low doses of DOP, significantly reduced levels of ALT, UA, and BUN were observed, suggesting DOP has a protective effect on liver and kidney function (Figures 4(a) and 4(b); Figures 5(a)–5(c)).

H&E staining showed that the liver tissue in the normal group had a normal architecture. The hepatic lobules were intact and clear, and the hepatocytes were arranged in a radial manner around the central portal vein. Most hepatocytes were polygonal with a rounded nucleus at the center of the cell; moreover, the nucleolus was clear, and the cytoplasm was uniform. In the PLGC group, hepatic lobules were abnormal, with swollen and degenerated hepatocytes exhibiting enlarged and at times even duel nuclei. Inflammatory cells, bile duct hyperplasia, and a significantly widened portal area were also observed. DOP treatment, however, resulted in hepatocytes showing no degeneration or swelling. What is more, these cells had round nuclei, with clear nucleoli and cytoplasm (Figure 4(c)).

H&E staining of the kidney showed that the renal glomeruli of the normal group had a normal structure devoid of congestion, necrosis, and inflammatory infiltration. Additionally, the basement membrane of the glomerulus appeared intact. The PLGC rat group, on the other hand, had obvious renal tubule epithelial cell edema, renal capsule stenosis, and epithelial cell shedding. All DOP treatment groups showed significant mitigation of those changes (Figure 5(d)).

### 3.6. Analysis of 8-OHdG in Serum

**Figure 6:**
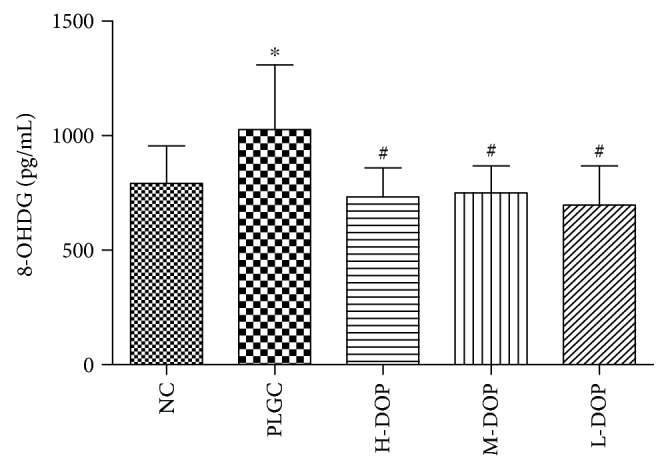
The effect of DOP on 8-OHdG in rat serum. ^∗^ *P* < 0.01 vs. normal group; ^#^ *P* < 0.05 vs. PLGC group.

Long-term drinking of MNNG in the model group caused the expression of 8-OHdG in plasma to increase significantly as compared to the normal control group (*P* < 0.05). On the contrary, treatment with high, medium, and low doses of DOP significantly reduced the expression of 8-OHdG as compared to control (*P* < 0.05) (Figure 6).

### 3.7. Immunohistochemical Analysis of NRF2

**Figure 7:**
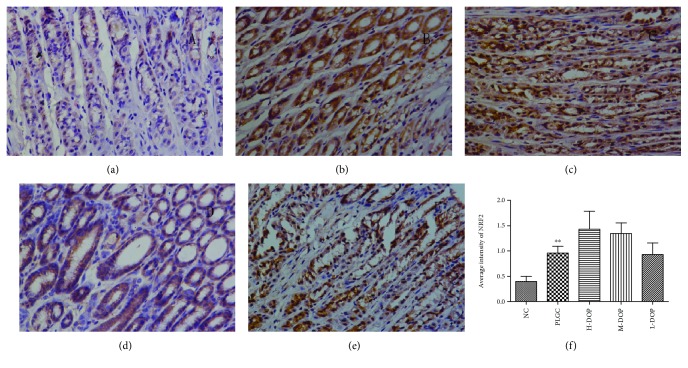
The effect of DOP on NRF2 expression by immunohistochemical analysis (×400; (a) normal, (b) PLGC, (c) H-DOP, (d) M-DOP, (e) L-DOP, (f) average intensity of NRF2. ^∗∗^ *P* < 0.01 vs. normal group; ^#^ *P* < 0.05 vs. PLGC group).

It was shown that the PLGC group saw significantly increased NRF2 expression compared to the normal control group (*P* < 0.01). PLGC treated with high levels of DOP (H-DOP) rendered NRF2 expression levels even higher, even when compared with the PLGC group (*P* < 0.05) (Figure 7).

### 3.8. Differential Protein Expression of NRF2 in Cytoplasmic and Nuclear Protein Fractions

**Figure 8:**
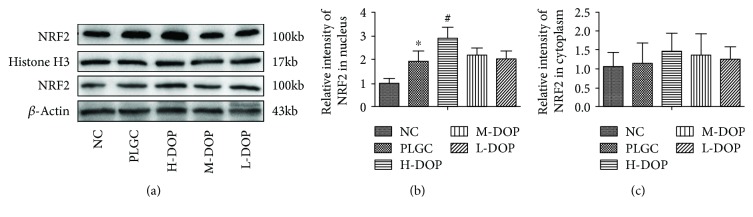
The effects of DOP on the expression of NRF2 protein in the nucleus and cytoplasm of gastric precancerous lesions in rats. ^∗^ *P* < 0.05 vs. normal group; ^#^ *P* < 0.05 vs. PLGC group.

As shown in Figure 8, protein NRF2 expression within the nuclei of the PLGC model increased significantly in comparison with the NC group (*P* < 0.05). Yet, the H-DOP group exhibited the greatest levels of nuclear NRF2 expression, significantly more abundant than even the PLGC model group (*P* < 0.05). However, cytoplasmic levels of NRF2 protein did not vary much between the PLGC model and the NC group. The H-DOP group, though, did report significantly different levels of cytoplasmic NRF2 compared to NC.

### 3.9. Total RNA Extraction and Quantitative Real-Time RT-PCR

**Figure 9:**
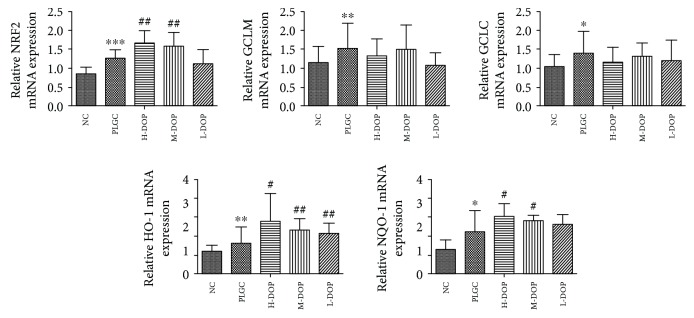
The gene expression in the NRF2 and its downstream antioxidant enzymes HO-1 and NQO-1 in rats. ^∗^ *P* < 0.05 and ^∗∗^ *P* < 0.01 vs. normal group; ^#^ *P* < 0.05 and ^##^ *P* < 0.01 vs. PLGC group.

The expression of NRF2 was increased in the PLGC model group, the H-DOP group, and the M-DOP group. This is most likely a result of the upregulated transcription of NRF2 mRNA. As a result of this increase, mRNA transcripts corresponding to downstream oxidases NQO-1 and HO-1 also increased significantly compared with the PLGC model group (*P* < 0.05, *P* < 0.01, *P* < 0.01; *P* < 0.05, *P* < 0.05). Yet, this did not affect the expression of either GCLC or GCLM (Figure 9).

### 3.10. Western Blot Analysis of NRF2, HO-1, and NQO-1 Expression in Rat Stomach Tissues

**Figure 10:**
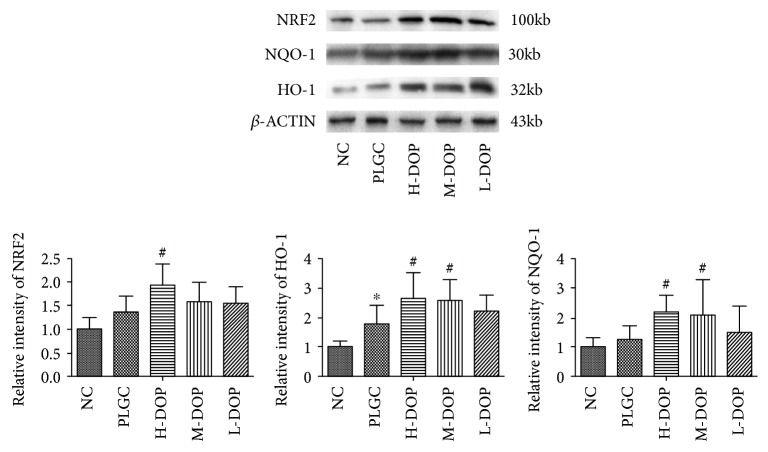
The protein expression of NRF2, HO-1, and NQO-1 in stomach tissues. ^∗^ *P* < 0.05 and ^∗∗^ *P* < 0.01 vs. normal group; ^#^ *P* < 0.05 and ^##^ *P* < 0.01 vs. PLGC group.

While protein expression of NRF2 and NQO-1 seemed to be elevated, tests showed no statistically significant variance compared to the PLGC group. While H-DOP increased NRF2 expression, the relative gray value was higher than that of the PLGC model group (*P* < 0.05). All doses of DOP resulted in more abundant expression of downstream proteins NQO-1 and HO-1, yet only the H-DOP and M-DOP groups reported a statistically significant increase (*P* < 0.05, *P* < 0.05; *P* < 0.05, *P* < 0.05) (Figure 10).

## 4. Discussion

Dendrobium officinale (DO), more commonly known as the dendrobium orchid herb, harbors a wide range of clinical benefits, including tumor resistance, immune support, and antiaging [28]. Moreover, it has a strong effect on nourishing the stomach Yin, playing an important role in treating gastrointestinal diseases. Zhang et al. reported that Dendrobium officinale polysaccharides could inhibit human gastric cancer cell in nude mice [22], while Zeng et al.'s study showed the polysaccharides of Dendrobium officinale Kimura et Migo could protect gastric mucosal cell against oxidative damage-induced apoptosis in vitro and in vivo [21]. Increased reactive oxygen species and oxidative stress injury may lead to gastric mucosal damage and malignancies, resulting in an increase of the DNA damage marker 8-OHdG [29, 30]. Our study is the first to investigate the capacity of DOP to inhibit PLGCs. The application of AB-PAS staining is widely used in the diagnoses of IM primarily because of its superiority to HE staining in cases of early detection [31]. We found that DOP decrease the degree of intestinal metaplasia and atypical hyperplasia while also protecting the gastric mucosa from damage. The data presented here show DOP to possess and inhibitory effect on PLGC, thus reducing the incidence of gastric carcinogenesis.

Transcription factor NRF2 is an active player in cancer chemoprevention and chemoresistance [32, 33]. Under conditions of oxidative stress, NRF2 is no longer sequestered by KEAP1 and is transferred to the nucleus, where it binds to the ARE and increases the expression of several antioxidant and phase II detoxification enzymes. This process ameliorates the damage that harmful substances might have on cells, playing a role in tumor prevention [11, 34]. Researchers have found that NRF2 expression is positively associated with aggressive tumor behavior in gastric cancer, suggesting NRF2 expression to be a potential indicator of poor prognoses in gastric cancer. This is somewhat due to its association with P-glycoprotein upregulation in gastric cancer [35–37]. Wang et al. reported that NRF2 and IGF-1 coexpression was highly elevated when transitioning from benign proliferative lesions to malignant lesions, showing significant differences between hyperplastic polyps, intraepithelial neoplasia, and adenocarcinoma [38]. In our study, prolonged exposure to MNNG induced genotoxicity and oxidative stress [39]. This was evidenced both by an increase in the levels of oxidative damage biomarker 8-OHdG and by upregulated expression of NRF2 in the PLGC model group, mirroring previously reported data [40]. After administration of DOP, levels of 8-OHdG decreased. Simultaneously, H-DOP saw significantly greater NRF2 expression in the gastric mucosa, as compared with the PLGC group. Upon further study, we observed nuclear accumulation of NRF2, with a marked increase in the nuclear expression of NRF2 protein in both the PLGC and the H-DOP group as compared with the NC group. However, NRF2 protein in the cytoplasm showed only a minimal increase in the PLGC model and DOP group. These results indicate DOP can increase the nuclear expression of NRF2 and perhaps upregulate enzymes downstream of NRF2.

As a master regulator of antioxidative stress, NRF2 regulates the expression of antioxidant genes and phase II detoxifying enzymes, such as HO-1 and NQO-1, both of which counteract the oxidative stress by facilitating the removal of ROS [41]. HO-1 is activated not only to protect oxidative injury but also to modulate the infiltrating inflammatory cells. It is directly implicated in the migration and invasion of cancer cells [42]. NQO-1 enzyme, on the other hand, stabilizes the *P*53 tumor suppressor against carcinogenesis [43]. It is reported that NQO-1-deficient mice showed reduced *P*53 induction and apoptosis, while simultaneously demonstrating increased susceptibility to chemically induced tumors [44]. In our study, MNNG induced DNA damage and oxidative stress, leading to increased protein and gene expression of HO-1 and NQO-1. Compared with the PLGC group, there is a marked increase in the expression of both HO-1 protein and RNA following DOP treatment. This study demonstrates NRF2 activated HO-1 in order to exert antioxidative and protective effects [45]. Compared with the PLGC group, NQO-1 mRNA and protein expression was upregulated in the H-DOP and M-DOP group.

GCLC and GCLM are both reported to be NRF2-dependent genes. GCLC is the rate-limiting enzyme for cellular GSH biosynthesis [46]. In this study, MNNG-induced PLGC was found to increase the expression of both GCLC mRNA and GCLM mRNA. However, DOP exposure had no additional effect, which suggests DOP to have no influence on GSH biosynthesis.

It is generally believed that, due to their pivotal role in drug metabolism, prolonged drug exposure will lead to hepatotoxicity and nephrotoxicity. Yet, Dendrobium officinale (DO) showed no toxicity or outstanding side effects even after broad clinical usage. In our study, we conducted histopathological examination and biochemical analysis of the liver and kidney. Serum ALT, UA, and BUN levels were increased in the model group. These levels decreased significantly following treatment with all experimental doses of DOP. The data reported here shows DOP to potentially harbor a protective effect on liver and kidney function.

## 5. Conclusion

In conclusion, our data introduces the novel idea that Dendrobium officinale polysaccharides (DOP) prevent precancerous lesions of gastric cancer (PLGC). In addition, we demonstrate DOP are capable of restraining the activity of 8-OHdG while increasing the nuclear expression of NRF2. All told, this activates downstream HO-1 and NQO-1 expression to improve antioxidant activity and protect gastric mucosal cells from oxidative damage. In additional, DOP can decrease serum levels of ALT, UA, and BUN, indicating DOP might protect liver and kidney function. These findings show DOP can be considered an effective healthcare product for the treatment of precancerous lesions of gastric cancer and perhaps someday play a critical role in combatting gastric cancer.

## Data Availability

The data used to support the findings of this study are included within the article.

## Supplementary Materials

Supplementary Materials Figure S1: standard curve of glucose and the content of DOP. Figure S2: the chromatogram of different molecular weight of dextran and the standard curve of dextran. Table S1: RT-PCR primer sequences and annealing temperature.
