# Supplementary material for: Dendrobium Officinale Polysaccharides Protect against MNNG-Induced PLGC in Rats via Activating the NRF2 and Antioxidant Enzymes HO-1 and NQO-1
Source: Oxid Med Cell Longev. 2019 Jun 4;2019:9310245. doi: 10.1155/2019/9310245 (PMC6589278; doi:10.1155/2019/9310245)
Supplement: Supplementary Materials — Figure S1: standard curve of glucose and the content of DOP. Figure S2: the chromatogram of different molecular weight of dextran and the standard curve of dextran. Table S1: RT-PCR primer sequences and annealing temperature. [file 9310245.f1.pdf]

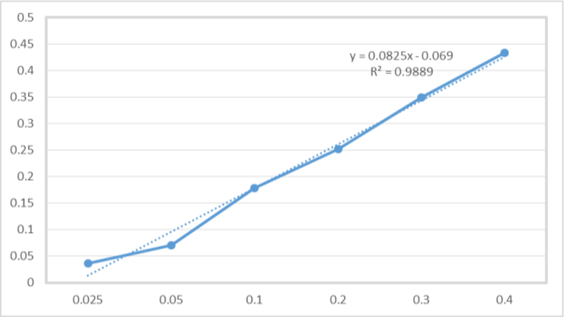

Sample	Content of DOP
Sample 1	76.59%
Sample 2	86.28%
Sample 3	86.41%

FIGURE S1 Standard curve of glucose and the content of DOP

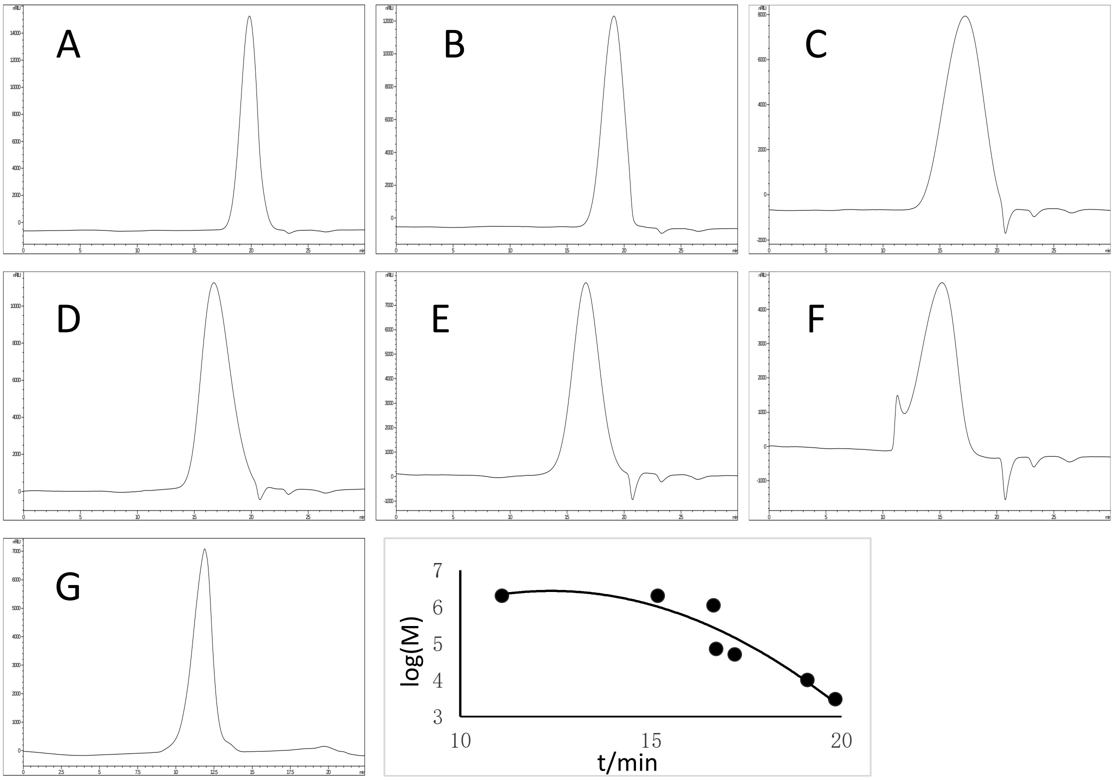

FIGURE S2 The chromatogram of different molecular weight of dextran and the Standard curve of dextran (A.3000; B.1 million C.5million; D.7 million; E.11 million; F.20 million G.200 million;)

Table S1 RT-PCR primer sequences and annealing temperature

Gene	Forward primer	Reverse primer
β-actin	CCCATCTATGAGGGTTACGC	TTTAATGTCACGCACGATTTC
KAEP1	CTGCATCCACCACAGCAGCGT	GTGCAGCACACAGACCCCGGC
NRF2	GCCCACATTCCCAAACAAGAT	CCAGAGAGCTATTGAGGGACTG
HO-1	AAGAGGCTAAGACCGCCTTC	GCATAAATTCCCACTGCCAC
NQO-1	TCACCACTCTACTTTGCTCCAA	TTTTCTGCTCCTCTTGAACCTC
Gclc	CCTCCTCCTCCAAACTCAGATA	CCACAAATACCACATAGGCAGA
Gclm	GCCACCAGATTTGACTGCCTTT	CAGGGATGCTTTCTTGAAGAGCTT
